# An In Vitro Evaluation of Robin’s Pincushion Extract as a Novel Bioactive-Based Antistaphylococcal Agent—Comparison to Rosehip and Black Rosehip

**DOI:** 10.3390/antibiotics13121178

**Published:** 2024-12-04

**Authors:** Olja Šovljanski, Milica Aćimović, Teodora Cvanić, Vanja Travičić, Aleksandra Popović, Jelena Vulić, Gordana Ćetković, Aleksandra Ranitović, Ana Tomić

**Affiliations:** 1 Faculty of Technology Novi Sad, University of Novi Sad, Bulevar Cara Lazara 1, 21000 Novi Sad, Serbia; oljasovljanski@uns.ac.rs (O.Š.); teodora.cvanic@uns.ac.rs (T.C.); vanjaseregelj@tf.uns.ac.rs (V.T.); jvulic@uns.ac.rs (J.V.); gcetkovic@uns.ac.rs (G.Ć.); 2 Institute of Field and Vegetable Crops Novi Sad, Maksima Gorkog 30, 21000 Novi Sad, Serbia; milica.acimovic@ifvcns.ns.ac.rs; 3 Faculty of Agriculture Novi Sad, University of Novi Sad, Trg Dositeja Obradovića 8, 21000 Novi Sad, Serbia; popovica@polj.uns.ac.rs

**Keywords:** *Rosa canina*, *Rosa spinosissima*, *Diplolepsis rosae*, rose gall wasp, antioxidant activity, antihyperglycemic, anti-inflammatory

## Abstract

**Introduction:** This study explores the bioactive properties of extracts obtained from Robin’s pincushion (*Diplolepis rosae*) collected in Sokobanja, Serbia. **Results:** Comprehensive in vitro assessments reveal high concentrations of total phenolics (186.37 mg GAE/g), along with significant levels of carotenoids (44.10 μg β-car/g). Robin’s pincushion exhibited superior antioxidant capacities across DPPH, ABTS, and reducing power assays, significantly outperforming comparable extracts from rosehip (*Rosa canina*) and black rosehip (*Rosa spinosissima*) in these activities. Additionally, high inhibitory effects were observed in antimicrobial assays, with the extract demonstrating minimal inhibitory concentrations (MIC) as low as 1.56 mg/mL against the *Staphylococcus* species. Notably, the extract achieved full bactericidal effect within 24 h in time-kill kinetic studies which additionally highlight its potent antistaphylococcal potential. **Materials and methods:** Analyzing their phytochemical profiles and evaluating their potential as antioxidant, anti-inflammatory, antihyperglycemic, and antimicrobial agents, wide-ranging evaluation of bioactivity of Robin’s pincushion was conducted. **Conclusions:** These findings highlight Robin’s pincushion as a promising natural source of bioactive compounds with potential applications in traditional and modern medicine for managing oxidative stress, inflammation, hyperglycemia, and microbial infections.

## 1. Introduction

Ethnopharmacological knowledge offers potential for the development of new pharmaceutical products [1]. This type of knowledge has been passed on in Serbia for a very long time, especially in rural areas of mountain regions [2,3,4,5,6,7,8,9]. Dog rose (*Rosa canina* L., Rosaceae) is mainly used as rosehip (*Cynosbati fructus*), a rich source of vitamin C, which is a long tradition spread worldwide. Apart from vitamin C, it contains other water-soluble vitamins such as K and B group (B1, B2 and B3), sugars, organic acids, pectins, flavonoids, tannins, carotenoids, fatty acids, macro- and microelements [10]. In Serbia, rosehip is used fresh as fruit for preparing juices, marmalade, and jams, while dried rosehip is used as favorable herbal tea in everyday life, as well as to prevent and treat colds and influenza [11]. According to numerous studies, rosehip extract expressed antioxidant, antimicrobial, antihyperglycemic, antihyperlipidemic and anticancer activities [12,13,14,15,16,17,18]. Due to its bioactivity, rosehip can be considered as a potential functional food as well as valuable raw material for the pharmaceutical industry [19].

In addition to rosehip, the edible fruits of the Burnet rose or Scotch rose (*Rosa spinosissima* L., syn. *R. pimpinellifolia* L.), commonly referred to as black rosehip, have been recognized since ancient times for their medicinal properties. These fruits have been traditionally used to treat respiratory and inflammatory conditions, as well as for their health-promoting benefits [20,21]. Today, this species is rarely used for medicinal or culinary purposes, despite its widespread distribution across a range of climates, from cold to temperate and continental regions of Europe and Siberia [22,23]. However, its distinctive phytochemical profile, rich in polyphenolic compounds, anthocyanins, carotenoids, and vitamins, positions it as an exceptional raw material with significant industrial potential for the development of nutraceutical and pharmaceutical products [21,24].

However, pests and diseases can reduce the quality and quantity of rosehip [25]. For example, as a response to damage induced by insects called rose gall wasp (*Diplolepis rosae* L., Hymenoptera: Cynipidae), plant galls develop, characterized by abnormal growths and deformed flowers [26]. In fact, overwinter females of gall wasps come out from galls in early spring and lay eggs on new flowers or leaf buds [27,28]. During development, insects produce multi-chambered galls, which look like bulges covered with thick yellow-green, chartreuse hair and can be found in sizes from 5 mm to 8 cm [29,30]. This species is particularly abundant in mountainous areas, often found near forests [31]. These formations are known as *Robin’s pincushion or* rose bedeguar gall. These galls are phytochemically distinct from the normal plant tissues, and over time humans have learned to use galls as therapeutics [26]. In Bulgarian traditional medicine, a decoction of Robin’s pincushion in water is used as an antitussive and anti-asthmatic remedy [32]. On the Iberian Peninsula, Robin’s pincushion is used as an eyewash for ocular inflammations, a mouthwash for gargling and toothache, and *a tisane for treating* kidney inflammation diseases [33].

There are no recorded written traces of *Robin’s pincushion* in traditional medicine in Serbia. However, local people from the Sokobanja region use this drug to treat inflammatory processes of the skin (called “*ružine šišarke*” in Serbian, literally translating to “*rose cones*” in English). Therefore, in this study, *Robin’s pincushion*, rosehip and black rosehip collected from the Sokobanja region (eastern part of Serbia), was analyzed to determine the content of total phenolics and carotenoids. Additionally, in vitro evaluation of antioxidant, anti-inflammatory, antihyperglycemic and antimicrobial activities was conducted, in order to provide scientific support for *Robin’s pincushion’s* application in traditional medicine and to compare obtained results with rosehip and black rosehip, whose flowers and fruits are parasitized by the rose gall wasp.

## 2. Results

### 2.1. The Phytochemical Composition and In Vitro Antioxidant Activity

The phytochemical analysis and antioxidant activity assays conducted on Robin’s pincushion, black rosehip, and rosehip extracts are summarized in Table 1. The results highlight Robin’s pincushion as an exceptionally rich source of bioactive compounds compared to the other tested extracts. Namely, the total phenolic content (TPh) was markedly higher in Robin’s pincushion extract, with a concentration of 186.37 mg GAE/g, compared to 9.65 mg GAE/g in rosehip and 7.24 mg GAE/g in black rosehip. These results indicate that Robin’s pincushion contains an abundance of phenolic compounds, which are recognized for their potent antioxidant properties. In contrast, rosehip and black rosehip exhibited significantly lower TPh values, suggesting comparatively reduced phenolic content. In terms of total carotenoid content (TCar), rosehip displayed the highest concentration at 62.24 μg β-car/g, followed by Robin’s pincushion with 44.10 μg β-car/g. Black rosehip contained only 8.10 μg β-car/g, indicating lower carotenoid levels. Despite rosehip having the highest carotenoid concentration, the combined phenolic and carotenoid profile of Robin’s pincushion suggests a distinct bioactive composition that may contribute to its potent antioxidant activity.

The antioxidant potential of the extracts was assessed through DPPH, ABTS, and reducing power (RP) assays, each providing insights into different mechanisms of antioxidant action. As can be seen in Table 1, Robin’s pincushion exhibited a DPPH radical scavenging activity of 152.07 mM TEAC/100 g, significantly outperforming rosehip and black rosehip, which showed values of 21.89 and 3.22 mM TEAC/100 g, respectively. The low IC50 value of the standard (Trolox at 0.14 ± 0.01 mM) underscores the strong radical scavenging ability of Robin’s pincushion, suggesting a high free radical neutralization capacity. In the ABTS assay, Robin’s pincushion again demonstrated superior activity, with an antioxidant capacity of 636.67 mM TEAC/100 g, compared to rosehip at 70.17 mM TEAC/100 g and black rosehip at 13.55 mM TEAC/100 g. The reference standard, Trolox, showed a significantly lower activity level (1.06 ± 0.04 mM TEAC), further emphasizing the high antioxidant potency of Robin’s pincushion. The reducing power of Robin’s pincushion extract was measured at 107.84 mM TEAC/100 mL, surpassing both rosehip (25.24 mM TEAC/100 mL) and black rosehip (3.4 mM TEAC/100 mL). This elevated RP value suggests that Robin’s pincushion has a strong electron-donating capacity, contributing to its overall antioxidant profile.

### 2.2. The HPLC Analyses of Polyphenolics

The High-Performance Liquid Chromatography (HPLC) analysis of polyphenolic compounds in Robin’s pincushion, black rosehip, and rosehip extracts identified substantial variations in phenolic profiles, as shown in Table 2. All chromatographs are presented in the Supplementary Figure S1. This analysis highlights Robin’s pincushion as a particularly rich source of polyphenols compared to the other extracts. Robin’s pincushion exhibited the highest concentration of p-hydroxybenzoic acid at 250.98 mg/100 g dw, significantly surpassing both rosehip (62.56 mg/100 g dw) and black rosehip (57.67 mg/100 g dw). This compound is known for its antioxidant properties, and its abundance in Robin’s pincushion suggests a strong potential for free radical scavenging. The presence of gallic acid was notable in Robin’s pincushion, with a concentration of 297.95 mg/100 g dw, while it was undetected in rosehip and present only at 16.89 mg/100 g dw in black rosehip. Known for its antimicrobial and antioxidant effects, gallic acid contributes to the enhanced bioactivity observed in Robin’s pincushion.

Robin’s pincushion contained a high level of protocatechuic acid (677.37 mg/100 g dw), compared to 15.96 mg/100 g dw in rosehip and 32.49 mg/100 g dw in black rosehip. This phenolic acid is recognized for its antioxidant and anti-inflammatory properties, further underscoring the superior bioactivity of Robin’s pincushion extract. Robin’s pincushion demonstrated an exceptionally high ellagic acid content of 1066.02 mg/100 g dw, whereas it was undetected in rosehip and observed at a lower concentration of 223.47 mg/100 g dw in black rosehip. Ellagic acid is a potent antioxidant and has been associated with anti-cancer properties, highlighting the therapeutic potential of Robin’s pincushion. This phenolic compound was found exclusively in Robin’s pincushion at 272.00 mg/100 g dw, while it was undetected in both rosehip and black rosehip extracts. Syringic acid has antioxidant and anti-inflammatory effects, contributing to the distinct bioactive profile of Robin’s pincushion. Both Robin’s pincushion and black rosehip contained high levels of vanillic acid, with concentrations of 392.03 mg/100 g dw and 404.19 mg/100 g dw, respectively, whereas rosehip contained only 21.12 mg/100 g dw. Vanillic acid is known for its antioxidant and antimicrobial properties, supporting the potential use of Robin’s pincushion in bioactive applications. Detected only in rosehip at a minimal concentration of 3.04 mg/100 g dw, ferulic acid was absent in both Robin’s pincushion and black rosehip. This low presence suggests that ferulic acid may not significantly contribute to the bioactivity of Robin’s pincushion. The total polyphenolic content in Robin’s pincushion extract reached 2956.35 mg/100 g dw, which is substantially higher than in black rosehip (754.23 mg/100 g dw) and rosehip (102.67 mg/100 g dw). This pronounced difference underscores the richness of Robin’s pincushion in bioactive polyphenols, which likely accounts for its superior antioxidant activity demonstrated in previous analyses.

### 2.3. Anti-Inflammatory and Antihyperglycemic Activities

The anti-inflammatory and antihyperglycemic properties of Robin’s pincushion, black rosehip, and rosehip extracts were evaluated, with results displayed in Table 3. These analyses highlight the potential therapeutic applications of Robin’s pincushion, especially in managing inflammation and hyperglycemia, as compared to the other tested extracts.

The anti-inflammatory activity (AIA) was measured as a percentage of inhibition, with Robin’s pincushion showing an AIA of 29.03%. This level is comparable to rosehip, which demonstrated an inhibition rate of 28.54%, but somewhat lower than black rosehip, which exhibited the highest anti-inflammatory activity at 46.31%. Despite this, Robin’s pincushion demonstrated considerably higher activity than the reference standard, acarbose, which showed an inhibition of only 1.14%. The relatively strong anti-inflammatory potential of Robin’s pincushion suggests its suitability as a natural agent for inflammation management, although black rosehip exhibited the highest efficacy among the extracts tested. In terms of antihyperglycemic activity (AHgA), Robin’s pincushion extract showed a remarkable inhibition rate of 96.30%, far surpassing both rosehip (31.67%) and black rosehip (27.84%). This inhibition rate is significantly higher than that of the standard, diclofenac sodium, which demonstrated only 0.001% inhibition. The high antihyperglycemic activity observed in Robin’s pincushion suggests its potential as a powerful natural agent for blood glucose regulation and a promising candidate for managing conditions like diabetes. This superior antihyperglycemic performance may be attributed to its high phenolic content, as polyphenols are known to positively influence glucose metabolism.

### 2.4. Antimicrobial Assessment—Determination of Inhibition Zones and Minimal Inhibitory Concentrations

The antimicrobial potential of Robin’s pincushion, black rosehip, and rosehip extracts was evaluated against several *Staphylococcus* species using the disk-diffusion method, with ceftazidime (10 µg/disc) as the reference standard (Table 4). Across all tested strains, Robin’s pincushion demonstrated substantial antimicrobial efficacy, often outperforming both rosehip and black rosehip extracts. Against *S. aureus*, Robin’s pincushion showed a prominent inhibition zone of 29.33 mm, significantly larger than those for rosehip (7.00 mm) and black rosehip (10.5 mm) and surpassing the 25 mm inhibition zone of ceftazidime. This suggests that Robin’s pincushion is highly effective against this bacterial strain.

For *S. saprophyticus*, the inhibition zone for Robin’s pincushion measured 24.00 mm, on par with the ceftazidime standard and larger than rosehip (7.00 mm) and black rosehip (14.00 mm). This finding highlights the comparable efficacy of Robin’s pincushion to conventional antibiotics in this context. In the case of *S. sciuri*, Robin’s pincushion displayed a large inhibition zone of 31.00 mm, far exceeding rosehip (7.00 mm) and black rosehip (18.00 mm) and approaching the ceftazidime zone of 28 mm, suggesting strong antimicrobial properties against this species. The efficacy of Robin’s pincushion was also observed against *S. epidermidis*, with an inhibition zone of 26.33 mm, matching that of ceftazidime (26 mm) and substantially higher than rosehip (7.00 mm) and black rosehip (16.5 mm). Finally, against *S. warneri*, Robin’s pincushion produced an inhibition zone of 29.00 mm, significantly larger than those of rosehip (7.00 mm) and black rosehip (12.00 mm), and nearly equal to ceftazidime (28 mm).

The antimicrobial efficacy of Robin’s pincushion, black rosehip, and rosehip extracts was further evaluated through minimal inhibitory concentration (MIC) assays against various *Staphylococcus* strains, with MIC values expressed in mg/mL (Table 5). These results underscore the potent inhibitory effects of Robin’s pincushion in comparison to black rosehip and rosehip, particularly against multiple *Staphylococcus* species.

For *S. aureus*, Robin’s pincushion exhibited a low MIC of 1.56 mg/mL, indicating strong bacteriostatic activity. In contrast, both rosehip and black rosehip extracts had MIC values greater than 50 mg/mL, suggesting considerably weaker or ineffective inhibitory capacity at tested concentrations. Against *S. saprophyticus*, Robin’s pincushion maintained a MIC of 1.56 mg/mL, while black rosehip and rosehip showed no inhibition below 50 mg/mL. This trend continued with *S. sciuri*, where Robin’s pincushion demonstrated a MIC of 3.125 mg/mL, considerably lower than black rosehip (25 mg/mL), highlighting its enhanced antimicrobial potential. Robin’s pincushion also inhibited *S. epidermidis* with a MIC of 12.5 mg/mL, outperforming black rosehip (25 mg/mL) and showing greater efficacy than rosehip. The lowest MIC values for Robin’s pincushion were consistently observed for *S. warneri*, with a MIC of 1.56 mg/mL, while rosehip and black rosehip were ineffective at the highest tested concentration of 50 mg/mL.

### 2.5. Pharmacodinamic Potential—Time-Kill Kinetics Study of Antimicrobial Effect

The pharmacodynamics potential or time-kill kinetics has been established to clarify the in vitro antimicrobial capacity of *Robin’s pincushion*. The time-kill kinetic study of Robin’s pincushion extract against various *Staphylococcus* strains provides insight into the bactericidal effects of the extract at its MIC concentration over a 48 h period (Table 6). This study demonstrates the rapid and effective action of Robin’s pincushion extract, with all tested *Staphylococcus* strains showing a substantial reduction in bacterial count within the initial hours, ultimately leading to complete bacterial eradication within 24 to 48 h.

For *S. aureus*, the bacterial count decreased from an initial concentration of 6.1 log CFU/mL to 4.9 log CFU/mL within 2 h. By the 6 h mark, the count had reduced further to 2.2 log CFU/mL, with a near-total reduction to 0.5 log CFU/mL by 12 h. Complete eradication (0.0 log CFU/mL) was observed at 18 h and maintained through the 48 h period, demonstrating the potent bactericidal effect of the extract. In the case of *S. saprophyticus*, the initial bacterial count of 6 log CFU/mL decreased gradually, reaching 3.6 log CFU/mL at 12 h and 1.3 log CFU/mL at 18 h, with complete elimination observed at 24 h. This gradual reduction followed by complete eradication indicates the effectiveness of the extract over an extended period, even with an initially slower decline. In the case of *S. sciuri*, an initial bacterial load of 6 log CFU/mL dropped to 5.7 log CFU/mL at 2 h, with a rapid decrease to 1.0 log CFU/mL by 12 h. Complete eradication was achieved by 18 h, showcasing the swift action of the extract in reducing bacterial viability in this strain. *S. epidermidis* showed a more gradual decrease, with bacterial counts dropping from 6.1 log CFU/mL to 5.8 log CFU/mL at 2 h and 2.7 log CFU/mL at 12 h. By 36 h, all bacterial counts reached 0.0 log CFU/mL, demonstrating the slower but ultimately effective action of the extract on this strain. For *S. warneri*, a rapid decline in bacterial count was observed, with an initial concentration of 5.9 log CFU/mL reduced to 4.1 log CFU/mL at 2 h and 1.7 log CFU/mL at 6 h. Complete elimination was achieved by 12 h, indicating a particularly effective bactericidal effect against this strain.

**Figure 1:**
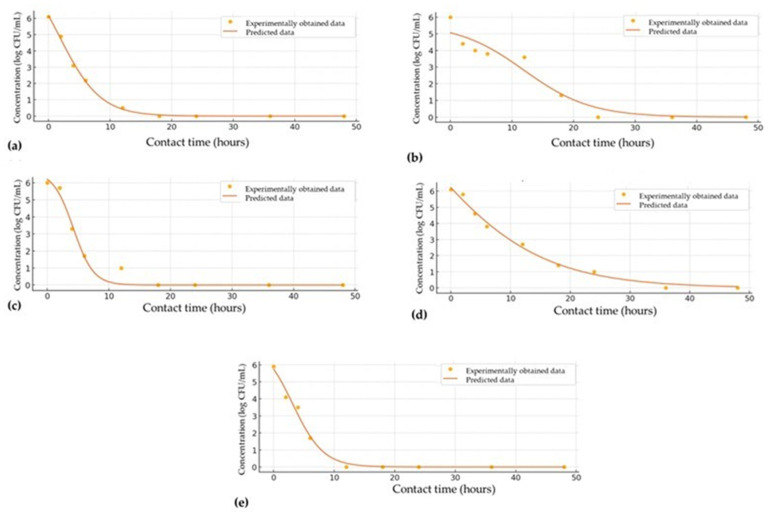
Time-kill kinetics study for (**a**) *S. aureus*; (**b**) *S. saprophyticus*; (**c**) *S. sciuri*; (**d**) *S. epidermidis*; and (**e**) *S. warneri* (dots indicate experimentally obtained data (see Table 6), while lines represent kinetic modeled values).

Figure 1 presents the time-kill kinetics of Robin’s pincushion extract against various *Staphylococcus* strains, with each subfigure illustrating the bacterial concentration (log CFU/mL) over time. The experimental data points (dots) align closely with the fitted kinetic model (lines), with kinetic parameters provided in Table 7.

In view of effect on *S. aureus* (Figure 1a), the extract demonstrated a rapid bactericidal effect, with the bacterial concentration dropping significantly within the first few hours and reaching complete eradication by 24 h. This swift reduction, as indicated by the kinetic parameters, highlights the potent antibacterial activity of Robin’s pincushion against *S. aureus*. For *S. saprophyticus* (Figure 1b), the reduction in bacterial concentration was more gradual, with a complete kill observed around the 48 h mark. The kinetic parameters for *S. saprophyticus* reflect a slower bactericidal action compared to *S. aureus* and *S. sciuri*. Similarly, *S. sciuri* (Figure 1c) showed a comparable rapid decline in bacterial load, with complete inhibition achieved within 24 h, suggesting a high rate constant and strong antibacterial efficacy against this strain as well. *S. epidermidis* (Figure 1d) also exhibited a moderate kill rate, with bacterial counts decreasing steadily over 36 h before reaching complete inhibition. The kinetic model for this strain indicates an intermediate rate constant, signifying moderate efficacy of the extract. *S. warneri* (Figure 1e) demonstrated rapid bacterial elimination similar to *S. aureus* and *S. sciuri*, with a swift decrease in bacterial load leading to complete eradication by 24 h. The kinetic parameters indicate a high rate constant for *S. warneri*, supporting the potent antimicrobial activity of Robin’s pincushion against this strain.

Table 7 provides an overview of the kinetic parameters and the “goodness of fit” for the kinetic models assessing the effect of Robin’s pincushion extract’s MIC values on different *Staphylococcus* strains. The kinetic model parameters, represented by the initial bacterial concentration (A) and the kill rate coefficient (k), along with verification parameters, indicate the model’s accuracy in capturing bacterial reduction over time.

The A values were similar across most strains, ranging from 5.76 log CFU/mL in *S. saprophyticus* to 6.52 log CFU/mL in *S. sciuri*. These values provide a baseline measure of the bacterial load prior to the bactericidal action of the extract. The k values, which reflects the rate of bacterial reduction, varied more significantly among the strains. *S. warneri* demonstrated the highest kill rate (0.19), followed closely by *S. sciuri* (0.17) and *S. aureus* (0.18), indicating rapid bacterial decline. In contrast, *S. saprophyticus* and *S. epidermidis* exhibited lower kill rate coefficients (0.08), suggesting a slower bactericidal response to the extract. The model’s fit accuracy was assessed using several statistical parameters. The reduced chi-square (*χ*^2^) values indicate the degree of alignment between observed and predicted data, with lower values signifying better fits. *S. aureus* exhibited the lowest *χ*^2^ value (0.119), indicating a highly accurate model fit, while *S. sciuri* had the highest χ^2^ (16.306), suggesting a weaker model fit for this strain. The root mean square error (RMSE) values further illustrate the model’s precision. *S. aureus* had the lowest RMSE (0.102), indicating minimal deviation between observed and predicted values. *S. saprophyticus* and *S. sciuri*, with higher RMSE values (0.530 and 0.358, respectively), reflect greater deviations in these model fits. Mean bias error (MBE) and mean percentage error (MPE) measure the model’s bias and average percentage deviation. Most strains had small MBE values, indicating minimal systematic error, though *S. sciuri* showed a notable negative MBE (−0.101), indicating a slight underestimation in model predictions. The MPE values were generally low, though *S. sciuri* had the highest negative MPE (−18.410), reflecting the model’s tendency to underestimate values for this strain. The coefficient of determination (*r*^2^), representing the proportion of variance explained by the model, was high across all strains, indicating a strong fit. *Staphylococcus aureus* showed the highest *r*^2^ (0.998), followed by *S. epidermidis* (0.992) and *S. warneri* (0.989), confirming excellent model fits. *S. saprophyticus* had a relatively lower r^2^ (0.939), indicating a less accurate fit compared to the other strains. Additional parameters, including skewness and kurtosis, provide insight into the distribution characteristics of model errors. Most strains exhibited slight positive skewness, indicating a tendency toward higher values, with S. saprophyticus showing near-zero skewness (−0.002), suggesting symmetry. Kurtosis values were negative for all strains, indicating platykurtic distributions with fewer extreme deviations than a normal distribution. The mean, standard deviation (SD), and variance of model predictions offer further context for error distribution. *S. epidermidis* had the highest mean prediction (2.822), indicating a higher central tendency, while *S. warneri* had the lowest mean (1.689). Variance values were relatively similar across strains, with *S. sciuri* showing the highest variance (5.384), suggesting more variability in model errors for this strain.

## 3. Discussion

The current study presents insight into the bioactive potential of Robin’s pincushion (*Diplolepis rosae*) extract, emphasizing applicability as natural agents in managing oxidative stress, staphylococcal-based infections, hyperglycemia, and inflammation. According to the obtained findings, the significant differences in bioactivity between Robin’s pincushion and traditional *Rosa canina* derivatives, including rosehip and black rosehip, highlights Robin’s pincushion as a particularly potent source of phytochemicals with strong therapeutic potential. Additionally, the examination of biological activities of common parts of the plants has been exhausted, and recently, galls or cecidia have become an interesting source with various biological activities. This is especially interesting because galling induces the host plant to secrete a wide range of phytochemicals, which in normal conditions would not happen, and it could be referred to metabolic re-routing of the plants. Taking into account that galls are usually called physiologic sinks due to the richness of secondary metabolites, it is not odd why investigating the biological activities of galls has attracted such great scientific attention. Some galls have been used as traditional medicines, especially galls from *Rhus*, *Pistacia*, *Quercus,* and *Terminalia* [26].

In this study, the phytochemical profile of Robin’s pincushion revealed high levels of phenolic compounds, measured at 186.37 mg GAE/g and 82.01 mg RE/g, respectively, as well as substantial quantities of carotenoids (44.10 μg β-car/g). These values notably exceed those found in rosehip and black rosehip, which exhibited comparatively lower concentrations. In antioxidant assays, Robin’s pincushion outperformed both rosehip and black rosehip across DPPH, ABTS, and reducing power evaluations, suggesting a robust ability to neutralize free radicals and prevent oxidative damage. The DPPH and ABTS radical scavenging activities, which were markedly higher for Robin’s pincushion, underscore its potential as a natural antioxidant comparable to synthetic antioxidants like Trolox. The superior antioxidant profile can likely be attributed to the high levels of phenolic acids identified, particularly protocatechuic acid, gallic acid, and ellagic acid, known for their potent free radical scavenging abilities. This enhanced antioxidant activity positions Robin’s pincushion as a candidate for applications in oxidative stress management, potentially benefiting chronic conditions such as cardiovascular diseases, diabetes, and neurodegenerative disorders. Although in the available literature it is possible to find the results of the biological activity of many galls, the information on *R. canina* galls is poor. The only data on the phytochemical content of *Robin’s pincushion* were found in the study of Coruh and Ercisli [27], which revealed that the content of phenolics for ten samples collected in Turkey varied between 66.34 and 93.35 mg GAE/g. It is noticeable that the *Robin’s pincushion* sample obtained in Serbia was richer in phenolics. Compared to rosehip fruit results, *Robin’s pincushion* has a higher content of phenolics according to Koczka et al. [34] (approximately 525 mg GAE/100 g) and Ercisli [35] (96 mg GAE/g). The same observation was noticed in this study with both spectrophotometrical and HPLC analyses, as Robin’s pincushion had higher polyphenol content than rosehip and black rosehip. According to Mohammadzadeh et al. [36], the total phenolic content of the *Quercus infectoria* galls extract was 16.21 mg GAE/g which showed lower amounts of these secondary metabolites compared with the tested *Robin’s pincushion*. Moreover, their phytochemical analysis confirmed that these extracts, besides phenolics and flavonoids, contain alkaloids, tannins, and saponins, and the combination of these secondary metabolites results in strong antibacterial effects. Furthermore, in the study of Azmaz et al. [37], the results of phenolic compounds for galls and their host plant extracts indicate that galls were richer in phenolics, reaching 479.56 mg GAE/g, while Coruh and Ercisli [27] reported that only 3 out of 10 had greater phenolic content. However, it is evident from the phytochemical compounds’ view that *Robin’s pincushion* is very rich in secondary metabolites. Unfortunately, no information on the antioxidant activity of *Robin’s pincushion* has been found. The obtained results of the *Robin’s pincushion* extracts express slightly higher DPPH activity compared to the *Rosa canina* L. extract whose DPPH activity was 127.8 µM TEAC/100 g [10], but significantly lower activity than in the work of Mihaylova et al. [38] (3.66 µM TEAC/g). However, the study of Basyigit et al. [39] revealed the DPPH and ABTS activities of *Quercus infectoria* galls were 2.29 mmol TEAC/g and 1.98 mmol TEAC/g, respectively, which showed lower activities than the tested sample. Furthermore, the DPPH assay has shown that the gall extract (IC50: 8.67 µg/mL) had stronger scavenging activity than ungalled leaf extracts (IC50: 54.37) of *Q. infectoria,* according to Azmaz et al. [37]. Therefore, it is noticeable that *Robin’s pincushion* extracts possess strong antioxidant activity. The obtained results for the anti-inflammatory activity of *Robin’s pincushion* at a concentration of 0.33 mg/mL showed that it was capable of inhibiting the denaturation of the proteins by 29.03%. Diclofenac sodium was used as a positive control, with an IC_50_ of 1.14 mg/mL. The antihyperglycemic activity was found to be less effective than the positive control, acarbose, with an inhibition of 96.30% achieved at a concentration of 10 mg/mL. The application of galls as folkloric medications has been practiced for decades, but now their relevance has been supported by biological assays as well. In the present study, the results confirmed the presence of secondary metabolites, for which other studies have already reported antimicrobial and antioxidant activity.

As can be seen in Table 3, Robin’s pincushion also demonstrated substantial anti-inflammatory and antihyperglycemic properties, with inhibition values of 29.03% for the anti-inflammatory assay and 96.3% for the antihyperglycemic assay. The antihyperglycemic activity, in particular, was notable, as it outperformed both rosehip and black rosehip by a significant margin. Phenolic compounds, particularly gallic acid and ellagic acid, are known for their ability to modulate inflammation and glucose metabolism [40,41], and their high presence in Robin’s pincushion likely contributes to these observed activities. Ellagic acid, found at 1066.02 mg/100 g in Robin’s pincushion, has been documented for its anti-diabetic effects and potential to inhibit α-glucosidase, an enzyme involved in carbohydrate digestion. The inhibition of this enzyme correlates with slower glucose absorption, which may help in managing postprandial blood glucose levels and is promising for developing natural antidiabetic agents. The anti-inflammatory potential of Robin’s pincushion, while not the highest among the tested extracts, remains significant when compared to the acarbose standard, which showed only minimal inhibition. This suggests that Robin’s pincushion could offer alternative or complementary benefits in managing inflammatory responses, particularly as chronic inflammation underpins various health conditions, including diabetes and cardiovascular disease. The moderate anti-inflammatory activity observed may complement its potent antioxidant and antihyperglycemic properties, which collectively contribute to its therapeutic potential.

The antimicrobial activity of Robin’s pincushion was profound, showing inhibition against several *Staphylococcus* species, including *S. aureus*, *S. saprophyticus*, *S. sciuri*, *S. epidermidis*, and *S. warneri*. The minimal inhibitory concentration (MIC) values ranged from 1.56 mg/mL for *S. aureus*, *S. saprophyticus*, and *S. warneri* to 12.5 mg/mL for *S. epidermidis*, demonstrating superior antibacterial efficacy compared to rosehip and black rosehip. The time-kill kinetic study further confirmed the bactericidal potential of Robin’s pincushion, with complete eradication of *S. aureus* and other *Staphylococcus* strains within 24 to 48 h. This rapid and effective bactericidal action aligns with previous studies, such as those on gall extracts from *Quercus infectoria*, where high phenolic content was similarly linked to potent antimicrobial activities. This efficacy against *Staphylococcus* strains is noteworthy given the increasing global challenge of antibiotic resistance. Plant-based antimicrobials, particularly those from unique sources like galls, provide promising alternatives to conventional antibiotics. The antimicrobial potential of Robin’s pincushion may be largely attributed to its polyphenolic content, especially ellagic acid, which has been reported to inhibit bacterial enzymes and disrupt cell membranes. Given that *Staphylococcus* species, including *S. aureus*, are implicated in skin infections and other clinical challenges, Robin’s pincushion could be explored further as a topical antimicrobial agent or adjunct treatment in antimicrobial-resistant infections.

## 4. Materials and Methods

### 4.1. Plant Material

**Figure 2:**
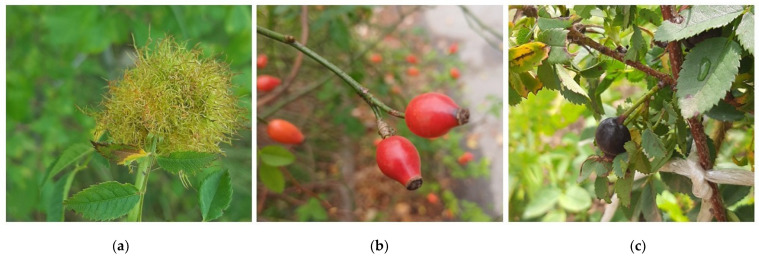
Samples used in this study: (**a**) *Robin’s pincushion* in nature; (**b**) rosehip; and (**c**) black rosehip (photo by Milica Aćimović).

*Robin’s pincushion, rosehips* and *black rosehips* (Figure 2) was collected during 2022 at localities near Sokobanja, Serbia. After the collecting, plant material was placed at ambient temperature, in a well-aerated place until constant weight. Voucher specimens were identified by PhD Milica Rat and deposited under Voucher Numbers 2-1193 (*Robin’s pincushion—rose gall*), 2-0046 (*rosehips—R. canina* L.) and 2-0017 (black rosehips—*R. spinosissima* L.) at the Herbarium BUNS, University of Novi Sad [42].

### 4.2. Sample Preparation and Extraction

The preparation of the sample included manual milling in a laboratory mortar and pestle (Lab Logistics Group GmbH, Meckenheim, Germany) until the coarse powder was obtained. *Robin’s pincushion*, rosehip, and black rosehip were extracted according to the procedure for analysis of total phenolic and carotenoid contents, as well as antioxidant and anti-inflammatory activity [43]. Summarily, the sample was extracted three times using an acetone/ethanol mixture (36:64) in a solid-to-solvent ratio of 1:20 for 10 min, with the same volume of solvents, on a laboratory shaker at 300 rpm, under light protection, at room temperature. For analysis of antihyperglycemic activity, the sample was extracted with a 50:50 ethanol/water mixture and potassium phosphate buffer, respectively, following the same steps of the extraction procedure. Extracts were evaporated and resuspended in saline solution for microbiological assays, as previously described by Šovljanski et al. [44].

### 4.3. HPLC Analysis

A Shimadzu Prominence chromatographic system (Shimadzu, Kyoto, Japan) was used to examine the prepared extracts. All separation conditions involved in the HPLC analysis are summarized in the Table 8. Namely, for particular substances, chromatograms were recorded at various wavelengths, typically for phenolic compounds. Separation was performed on a Luna C-18 RP column, 5 mm, 250 mm × 4.6 mm with a C18 guard column, 4 mm × 30 mm (both from Phenomenex, Torrance, CA, USA) and analyzed by the Diode Array Detector SPD-M20A (Shimadzu, Kyoto, Japan). At a flow rate of 1 mL/min, two mobile phases—A (acetonitrile) and B (1% formic acid)—were employed, and their gradient profiles looked like the following: 0–10 min from 10 to 25% B, 10–20 min linearly increasing to 60% B, and 20–30 min linearly increasing to 70% B, then 10 min reverting to the starting 10% B with an extra 5 min of equilibration time. The results are reported as mg/100 g sample dry weight (dw).

The phytochemicals were quantified using calibration curves prepared with authentic standards. The concentrations of phenolic compounds were expressed as milligrams of compound per 100 g of sample dry weight (dw). This approach ensured accurate identification and quantification of polyphenolic compounds in the extracts.

### 4.4. Determination of Phytochemical Composition

The phytochemical compositions of all three samples (Robin’s pincushion, rosehip, and black rosehip) were estimated by a few spectrophotometric methods adapted to the microscale to obtain information about the content of total phenolics, and carotenoids. Total phenolic content (TPh) was determined using the Folin–Ciocalteau reagents, as described in the work of Tumbas-Šaponjac et al. [45]. The results for the total content of phenolics were expressed as gallic acid equivalents (GAE) per 1 g of sample. Furthermore, the total carotenoids (TCar) content was determined and expressed as μg of β-carotene equivalents (β-car) per 1 g of a sample using the method described by Šovljanski et al. [46].

### 4.5. Determination of Antioxidant Activity

Three in vitro antioxidant assays’ composition of all three samples (Robin’s pincushion, rosehip, and black rosehip) were used to determine the antioxidant activity: 2,2-diphenyl-1-picrylhydrazyl (DPPH), 2,20-azino-bis-3-ethylbenzothiazoline-6-sulphonic acid (ABTS) and reducing power (RP) stated in work of Šovljanski et al. [46]. The results were expressed as micromoles of Trolox equivalent (TE) per 100 g of sample.

### 4.6. Determination of Anti-Inflammatory and Antihyperglycemic Activity

The anti-inflammatory and antihyperglycemic assays’ compositions of all three samples (Robin’s pincushion, rosehip, and black rosehip) were performed following the methods outlined in Ranitović et al. [47]. For the anti-inflammatory assay, which evaluates the sample’s ability to inhibit protein denaturation in egg albumin, three samples were prepared: Robin’s pincushion at a concentration of 0.33 mg/mL, and rosehip and black rosehip at 25 mg/mL. In the antihyperglycemic assay, which assesses the inhibition potential of α-glucosidase (AHgA), concentrations were adjusted to 10 mg/mL for all samples. Both assays were conducted using in vitro spectrophotometric methods, with results expressed as percentages (%).

### 4.7. Antimicrobial Potential

To investigate antimicrobial activity of all three samples (Robin’s pincushion, rosehip, and black rosehip), *Staphylococcus aureus* ATCC 11632, *S. sciuri* St2, *S. saprophyticus* St1, *S. epidermidis* St7, and *S. warneri* St8 were employed. All used cultures are part of the Collection of the microorganisms at Laboratory of microbiology at Faculty of Technology Novi Sad. The overnight cultures were prepared as follows: (1) the bacteria were streaked from −80 °C glycerol stock onto Müller-Hinton Agar (MHA, HiMedia, Mumbai, India) and incubated aerobically at 37 °C for 48 h. The examination of the antimicrobial potential of *R. canina* galls was performed in two steps: preliminary investigation by using the disk diffusion method and determination of minimal inhibitory concentration (MIC) by the dilution method. Both methods have been earlier described in detail by Pavlić et al. [48]. All tests have been carried out in triplicate, while the positive control for the disk-diffusion test was Oxoid™ Ceftazidime Antimicrobial Susceptibility discs (10 µg/disc, ThermoFisher, Waltham, MA, USA). The pharmacodynamic potential of antimicrobial activity was carried out by following the recommendations of Aćimović et al. [49]. The microorganisms that showed antimicrobial potential (approx. 10^6^ CFU/mL) have been subjected to MIC concentrations of the individual extract. In defined time intervals (0, 2, 4, 6, 12, 18, 24, 36, and 48 h), the samples were taken, and bacterial concentration was determined by streaking onto MHA plates. This study aimed to analyze the Minimum Inhibitory Concentration (MIC) effect decay across five bacterial species. The analysis was conducted in several steps using a sigmoidal kinetic model, with parameters estimated through non-linear regression. The fitted parameters were used to understand the time-kill characteristics of each species. Through time-kill kinetics, the real activity of the substances can be quantified as antimicrobials as a function of contact time between the cells and the targeted concentration of the examined substance.

Contact time (*t*) points (0, 2, 4, 6, 12, 18, 24, 36, and 48 h) and MIC effect values for each species were extracted and structured for modeling. A sigmoidal model was applied to describe the MIC decay over time, defined as follows:

(1) $$X(t)=A\times e^{-k\times t}$$

where X(*t*) represents bacterial concentration over contact time, A represents the calculated initial value of bacterial concentration, while *k* is the kill rate constant.

## 5. Conclusions

This study establishes Robin’s pincushion as a potent natural source of bioactive compounds with remarkable antioxidant, antimicrobial, antihyperglycemic, and moderate anti-inflammatory activities. The findings underscore its promise in therapeutic applications, positioning Robin’s pincushion as an alternative bioactive agent with applications spanning traditional and modern medicine. The unique phytochemical profile induced by gall formation sets it apart from conventional plant extracts, making it a valuable candidate for further exploration in bioactive compound research and development. This study highlights new data on *Robin’s pincushion* extracts. The results support the use of gall extracts as promising sources of potential antioxidants that may be effective as antimicrobial agents, due to the presence of secondary metabolites and expressed antioxidant activity. This study has found that *Robin’s pincushion* shows significant antifungal activity and moderate antibacterial activity. In further work, it would be interesting to know more precisely the phytochemical content to better understand what is responsible for the antimicrobial properties of *Robin’s pincushion*. Also, with a better understanding of this annual plant tissue, it is easier to expand its field of potential use.

Comparing Robin’s pincushion with rosehip and black rosehip highlights its distinct phytochemical composition and enhanced bioactivity across multiple assays. The gall formation process appears to induce a unique biochemical pathway that amplifies the production of secondary metabolites, including polyphenols, which likely accounts for its superior bioactivity. The gall formation, a plant’s response to insect-induced stress, may lead to a re-routing of metabolic processes that favors the synthesis of bioactive compounds typically limited in normal plant tissues. This metabolic adaptation results in a profile rich in antimicrobial and antioxidant compounds, reinforcing the relevance of galls as a valuable bioactive resource.

The results from this study highlight Robin’s pincushion’s potential as a useful bioactive agent suitable for multiple therapeutic applications. Future studies could benefit from isolating specific bioactive compounds in Robin’s pincushion to further delineate their individual contributions to antioxidant, antimicrobial, and antihyperglycemic activities. Moreover, expanding the spectrum of pathogens tested and assessing the in vivo efficacy could provide more insights into its potential clinical applications. Investigating its effects in wound healing, anti-aging formulations, or as an adjunct to diabetic care could pave the way for developing novel, plant-based treatments. Additionally, exploring the cultivation and sustainable harvesting of Robin’s pincushion in regions where it naturally occurs could support its use in both traditional medicine and modern pharmaceuticals. By optimizing extraction techniques and identifying scalable processing methods, Robin’s pincushion extracts could be integrated into nutraceutical and pharmaceutical products aimed at managing oxidative stress, infections, and metabolic disorders.

## Figures and Tables

**Table 1:** Phytochemical composition expressed as total phenolic (TPh) and total carotenoid (TCar) content and in vitro antioxidant activity via three different tests of Robin’s pincushion, rosehip and black rosehip extracts.

		Units	Robin’s Pincushion	Rosehip	Black Rosehip	Standard
Phytochemical composition	TPh	mg GAE/g	186.37 ± 12.21 ^a^	9.65 ± 0.28 ^b^	7.24 ± 0.18 ^b^	/
	TCar	μg β-car/g	44.10 ± 0.99 ^b^	62.24 ± 0.40 ^a^	8.10 ± 0.74 ^c^	/
Antioxidant activity	DPPH	mM TEAC/100 g	152.07 ± 5.19 ^a^	21.89 ± 0.87 ^b^	3.22 ± 0.13 ^c^	0.14 ± 0.01 ^d^
	ABTS		636.67 ± 19.32 ^a^	70.17 ± 2.05 ^b^	13.55 ± 0.45 ^c^	1.06 ± 0.04 ^d^
	RP		107.84 ± 3.56 ^a^	25.24 ± 1.59 ^b^	3.4 ± 0.04 ^c^	0.12 ± 0.02 ^d^

Means in the same column with different superscripts are statistically different (*p* < 0.05; Tukey HSD post-hoc test); standard compound in the antioxidant assays was Trolox.

**Table 2:** The HPLC analyses of polyphenolics for Robin’s pincushion, black rosehip, and rosehip extracts.

Phenols (mg/100 g dw)	Robin’s Pincushion	Rosehip	Black Rosehip
p-Hydroxybenzoic acid	250.98 ± 0.00 ^a^	62.56 ± 0.01 ^b^	57.67 ± 0.00 ^b^
Gallic acid	297.95 ± 0.01 ^a^	nd	16.89 ± 0.00 ^b^
Protocatechin acid	677.37 ± 0.00 ^a^	15.96 ± 0.00 ^b^	32.49 ± 0.00 ^b^
Ellagic acid	1066.02 ± 0.02 ^a^	nd	223.47 ± 0.01 ^b^
Syringic acid	272.00 ± 0.00 ^a^	nd	19.53 ± 0.00 ^b^
Vanillic acid	392.03 ± 0.00 ^a^	21.12 ± 0.00 ^b^	404.19 ± 0.01 ^a^
Ferulic acid	nd	3.04 ± 0.00 ^a^	nd
Total phenols	2956.35 ± 0.03 ^a^	102.67 ± 0.01 ^b^	754.23 ± 0.02 ^c^

Means in the same column with different superscripts are statistically different (*p* < 0.05; Tukey HSD post-hoc test); nd—not detected.

**Table 3:** The anti-inflammatory (AIA) and antihyperglycemic (AHgA) activities of Robin’s pincushion, rosehip, and black rosehip extracts expressed as % of inhibition.

Analyses	Robin’s Pincushion	Rosehip	Black Rosehip	Standard (IC50)
AIA	29.03 ± 1.28 ^b^	28.54 ± 0.01 ^b^	46.31 ± 0.12 ^a^	1.14 ± 0.03 ^c^
AHgA	96.30 ± 0.62 ^a^	31.67 ± 0.44 ^b^	27.84 ± 0.37 ^b^	0.001 ± 0.00 ^c^

Means in the same column with different superscripts are statistically different (*p* < 0.05; Tukey HSD post-hoc test)*;* IC50—values of used standard compounds in the bioactivity assays: for AIA it was acarbose, while for AHgA it was diclofenac sodium.

**Table 4:** Disk-diffusion test for Robin’s pincushion, black rosehip, and rosehip extracts expressed as inhibition zone in mm.

Test Bacteria	Robin’s Pincushion	Rosehip	Black Rosehip	Antibiotic Control
*Staphylococcus aureus*	29.33 ± 0.56 ^a^	7.00 ± 0.00 ^d^	10.5 ± 0.56 ^c^	25 ± 0.00 ^b^
*S. saprophyticus*	24.00 ± 0.00 ^a^	7.00 ± 0.00 ^c^	14.00 ± 0.00 ^b^	24 ± 1.00 ^a^
*S. sciuri*	31.00 ± 1.00 ^a^	7.00 ± 0.00 ^d^	18.00 ± 1.00 ^c^	28 ± 0.00 ^b^
*S. epidermidis*	26.33 ± 0.56 ^a^	7.00 ± 0.00 ^c^	16.5 ± 0.33 ^b^	26 ± 0.00 ^a^
*S. warneri*	29.00 ± 0.00 ^a^	7.00 ± 0.00 ^c^	12.00 ± 1.00 ^b^	28 ± 0.00 ^a^

Means in the same column with different superscripts are statistically different (*p* < 0.05; Tukey HSD post-hoc test)*;* antibiotic control was ceftazidime (10 µg/disc).

**Table 5:** Minimal inhibitory concentrations (MICs) for Robin’s pincushion, rosehip, and black rosehip extracts.

Test Bacteria	Robin’s Pincushion	Rosehip	Black Rosehip
*Staphylococcus aureus*	1.56 ± 0.00	>50*	>50
*S. saprophyticus*	1.56 ± 0.00	>50	>50
*S. sciuri*	3.125 ± 0.00	>50	25 ± 0.00
*S. epidermidis*	12.5 ± 0.00	>50	25 ± 0.00
*S. warneri*	1.56 ± 0.00	>50	>50

* as the initial value of extracts was used at a concentration of 50 mg/mL.

**Table 6:** Time-kill kinetic study experimentally obtained results for contact between MIC value of Robin’s pincushion extract and *Staphylococcus* strains expressed as contact time in hours.

Test Bacteria	0	2	4	6	12	18	24	36	48
*S. aureus*	6.1 ± 0.1	4.9 ± 0.1	3.1 ± 0.2	2.2 ± 0.2	0.5 ± 0.1	0.0 ± 0.0	0.0 ± 0.0	0.0 ± 0.0	0.0 ± 0.0
*S. saprophyticus*	6 ± 0.0	4.4 ± 0.0	4 ± 0.3	3.8 ± 0.0	3.6 ± 0.3	1.3 ± 0.2	0.0 ± 0.0	0.0 ± 0.0	0.0 ± 0.0
*S. sciuri*	6 ± 0.3	5.7 ± 0.0	3.3 ± 0.2	1.7 ± 0.0	1.0 ± 0.1	0.0 ± 0.0	0.0 ± 0.0	0.0 ± 0.0	0.0 ± 0.0
*S. epidermidis*	6.1 ± 0.1	5.8 ± 0.1	4.6 ± 0.0	3.8 ± 0.0	2.7 ± 0.2	1.4 ± 0.1	1.0 ± 0.2	0.0 ± 0.0	0.0 ± 0.0
*S. warneri*	5.9 ± 0.0	4.1 ± 0.2	3.5 ± 0.1	1.7 ± 0.0	0.0 ± 0.0	0.0 ± 0.0	0.0 ± 0.0	0.0 ± 0.0	0.0 ± 0.0

**Table 7:** The kinetic parameters and “goodness of the fit” for kinetic models obtained for MIC values’ effect on the *Staphylococcus* strain.

	Kinetic Models Parameters		Verification Parameters									
Test Bacteria	A (log CFU/mL)	k	*χ* ^2^	RMSE	MBE	MPE	*r* ^2^	Skew.	Kurt.	Mean	SD	Var.
*S. aureus*	6.36	0.18	0.119	0.102	0.003	−2.756	0.998	0.778	−0.914	1.867	2.225	4.951
*S. saprophyticus*	5.76	0.08	1.181	0.530	0.046	0.731	0.939	−0.002	−1.456	2.567	2.139	4.573
*S. sciuri*	6.52	0.17	16.306	0.358	−0.101	−18.410	0.976	0.789	−0.996	1.967	2.320	5.384
*S. epidermidis*	6.36	0.08	0.438	0.204	0.021	−1.968	0.992	0.142	−1.459	2.822	2.236	5.002
*S. warneri*	6.07	0.19	0.351	0.221	0.023	0.629	0.989	0.804	−0.869	1.689	2.137	4.565

A—predicted initial values of bacterial concentration; k—kill rate coefficient; χ^2^—reduced chi-square; RMSE—root mean square error; MBE—mean bias error; MPE—mean percentage error; *r*^2^—coefficient of determination; Skew.—skewedness; Kurt.—kurtosis; SD—standard deviation; and Var.—variance.

**Table 8:** HPLC method summary.

Parameter	Condition
Instrument	Shimadzu Prominence chromatographic system
Column	Luna C-18 RP column, 5 μm, 250 mm × 4.6 mm with C18 guard column (4 mm × 30 mm)
Detector	Diode Array Detector SPD-M20A
Flow rate	1 mL/min
Mobile phase A	Acetonitrile
Mobile phase B	1% Formic acid
Gradient profile	0–10 min: 10–25% B; 10–20 min: 25–60% B; 20–30 min: 60–70% B; 30–40 min: return to 10% B and 5 min equilibration
Detection wavelength	240 nm or/and 260 nm

## Data Availability

The original contributions presented in the study are included in the article, further inquiries can be directed to the corresponding authors.

## Supplementary Materials

The following supporting information can be downloaded at: https://www.mdpi.com/article/10.3390/antibiotics13121178/s1, Supplementary Figure S1. HPLC chromatographs of extracts of (a) Robin’s pincushion (240 nm); (b) rosehip (240 nm); (c) rosehip (260 nm); (d) black rosehip (240 nm); and (e) black rosehip (260 nm).

## References

[1] Pirintsos S., Panagiotopoulos A., Bariotakis M., Daskalakis V., Lionis C., Sourvinos G., Karakasiliotis I., Kampa M., Castanas E. (2022). From traditional ethnopharmacology to modern natural drug discovery: A methodology discussion and specific examples. Molecules.

[2] Janaćković P., Gavrilović M., Miletić M., Radulović M., Kolašinac S., Stevanović Z.D. (2022). Small regions as key sources of traditional knowledge: A quantitative ethnobotanical survey in the central Balkans. J. Ethnobiol. Ethnomed..

[3] Jarić S., Mačkukanović-Jocić M., Đurđević L., Mitrović M., Kostić O., Karadžić B., Pavlović P. (2015). An ethnobotanical survey of traditionally used plants on Suva planina mountain (south-eastern Serbia). J. Ethnopharmacol..

[4] Jarić S., Popović Z., Mačukanović-Jocić M., Đurđević L., Mijatović M., Karadžić B., Mitrović M., Pavlović P. (2007). An ethnobotanical study on the usage of wild medicinal herbs from Kopaonik Mountain (Central Serbia). J. Ethnopharmacol..

[5] Matejić J., Stefanović N., Ivković M., Živanović N., Marin P., Džamić A. (2020). Traditional uses of autochtonus medicinal and ritual plants and other remedies for health in Eastern and South-Eastern Serbia. J. Ethnopharmacol..

[6] Šavikin K., Zdunić G., Menković N., Živković J., Ćujić N., Tereščenko M., Bigović D. (2013). Ethnobotanical study on traditional use of medicinal plants in South-Western Serbia, Zlatibor district. J. Ethnopharmacol..

[7] Živković J., Ilić M., Šavikin K., Zdunić G., Ilić A., Stojković D. (2020). Traditional use of medicinal plants in south-eastern Serbia (Pčinja district): Ethnopharmacological investigation on the current status and comparison with half a century old data. Front. Pharmacol..

[8] Živković J., Ilić M., Zdunić G., Jovanović-Lješković N., Menković N., Šavikin K. (2021). Traditional use of medicinal plants in Jablanica district (South-Eastern Serbia): Ethnobotanical survey and comparison with scientific data. Genet. Resour. Crop Evol..

[9] Zlatković B., Bogosavljević S., Radivojević A., Pavlović M. (2014). Traditional use of the native medicinal plant resource of Mt. Rtanj (Eastern Serbia): Ethnobotanical evaluation and comarison. J. Ethnopharmacol..

[10] Roman I., Stănilă A., Stănilă S. (2013). Bioactive compounds and antioxidant activity of *Rosa canina* L. biotypes from spontaneous flora of Transylvania. Chem. Cent. J..

[11] Marković M., Pljevljakušić D., Nikolić B., Rakonjac L. (2020). Application of dog rose (*Rosa canina* L.) in ethnomedicine of the Pirot County. Pirot. Zb..

[12] Saaby L., Jäger A.K., Moesby L., Hansen E.W., Christensen S.B. (2010). Isolation of immunomodulatory triterpene acids from a standardized rose hip powder (*Rosa canina* L.). Phytother. Res..

[13] Jiménez S., Gascón S., Luquin A., Laguna M., Ancin-Azpilicueta C., Rodríguez-Yoldi M.J. (2016). *Rosa canina* extracts have antiproliferative and antioxidant effects on Caco-2 human colon cancer. PLoS ONE.

[14] Taghizadeh M., Rashidi A.A., Taherian A.A., Vakili Z., Sajadian M.S., Ghardashi M. (2016). Antidiabetic and antihyperlipidemic effects of ethanol extract of *Rosa canina* L. fruit on diabetic rats: An experimental study with histopathological evaluations. J. Evid. Based Complement. Altern. Med..

[15] Vlaicu P.A., Untea A.E., Turcu R.P., Panaite T.D., Saracila M. (2022). Rosehip (*Rosa canina* L.) meal as a natural antioxidant on lipid and protein quality and shelf-life of polyunsaturated fatty acids enriched eggs. Antioxidants.

[16] Paunović D., Kalušević A., Petrović T., Urošević T., Djinović D., Nedović V., Popović-Djordjević J. (2019). Assessment of chemical and antioxidant properties of fresh and dried rosehip (*Rosa canina* L.). Not. Bot. Horti Agrobot..

[17] Kilinc K., Demir S., Turan I., Mentese A., Orem A., Sonmez M., Aliyazicioglu Y. (2019). *Rosa canina* extract has antiproliferative and proapoptotic effects on human lung and prostate cancer cells. Nutr. Cancer.

[18] Rovná. K., Ivanišová E., Žiarovská J., Ferus P., Terentjeva M., Kowalczewski P.Ł., Kačániová M. (2020). Characterization of *Rosa canina* fruits collected in urban areas of Slovakia. Genome size, iPBS profiles and antioxidant and antimicrobial activities. Molecules.

[19] Latinović S., Brkljača M., Vujasin M., Kukrić Z., Odžaković B. (2020). The potential bioactivity of the wild grown rosehip (*Rosa canina* L.) and pomegranate (*Punica granatum* L.). Adv. Technol..

[20] Andersson S.C., Rumpunen K., Johansson E., Olsson M.E. (2011). Carotenoid content and composition in rose hips (*Rosa* spp.) during ripening, determination of suitable maturity marker and implications for health promoting food products. Food Chem..

[21] Findik B.T., Yildiz H., Akdeniz M., Yener I., Yilmaz M.A., Cakir O., Ertas A. (2024). Phytochemical profile, enzyme inhibition, antioxidant, and antibacterial activity of *Rosa pimpinellifolia* L.: A comprehensive study to investigate the bioactivity of different parts (whole fruit, pulp, and seed part) of the fruit. Food Chem..

[22] Mayland-Quellhorst E., Foller J., Wissemann V. (2012). Biological Flora of the British Isles: *Rosa spinosissima* L. J. Ecol..

[23] Žarković L.D., Stanković S.S., Veljić M.M., Marin P.D., Džamić A. (2022). Flower micromorphology of eight wild-growing *Rosa* species (*Rosaceae*) from Serbia. Biologia.

[24] Odabas H.I., Koca I. (2021). Simultaneous separation and preliminary purification of anthocyanins from *Rosa pimpinellifolia* L. fruits by microwave assisted aqueous two-phase extraction. Food Bioprod. Process..

[25] Bozhuyuk M.R., Ercisli S., Karatas N., Ekiert H., Elansary H.O., Szopa A. (2021). Morphological and biochemical diversity in fruits of unsprayed *Rosa canina* and *Rosa dumalis* ecotypes found in different agroecological conditions. Sustainability.

[26] Patel S., Rauf A., Khan H. (2018). The relevance of folkloric usage of plant galls as medicines: Finding the scientific rationale. Biomed. Pharmacother..

[27] Coruh S., Ercisli S. (2010). Interactions between galling insects and plant total phenolic contents in *Rosa canina* L. Sci. Res. Essays.

[28] Laszlo Z., Tothmeresz B. (2011). Parasitoids of the bedeguar gall (*Diplolepis rosae*): Effect of host scale on density and prevalence. Acta Zool. Acad. Sci. Hung..

[29] Mete O., Mergen O. (2016). The community members associated with rose gall wasp *Diplolepis fructuum* (Rübsaamen, 1895) (Hymenoptera: Cynipidae) in Tokat Province of Turkey. Turk. J. Zool..

[30] Todorov I., Boyadzhiev P., Antov M., Stojanova A. (2022). Correction to: Interrupted hibernation of the gall-inducer affects its parasitoids—A case study on some gall communities of *Diplolepis rosae* (Hymenoptera: Cynipidae) in Bulgaria. Biologia.

[31] Sardón-Gutiérrez S., Gil-Tapetado D., Gómez J., Nieves-Aldrey J.L. (2021). Ecological niche modelling of species of the rose gall wasp Diplolepis (Hymenoptera: Cynipidae) on the Iberian Peninsula. Eur. J. Entomol..

[32] Kozuharova E., Benbassat N., Napier J. (2012). New records of the remedial properties of vascular plants, some traditionally accepted as medicinal plants and some less familiar to ethnobotanists. Phytol. Balc..

[33] Agelet A., Valles J. (2003). Studies on pharmacological ethnobotany in the region of Pallars (Pyrinees, Catalonia, Iberian Peninsula). Part II. New or very rare uses of previously known medicinal plants. J. Ethnopharmacol..

[34] Koczka N., Stefanovits-Bányai É., Ombódi A. (2018). Total polyphenol content and antioxidant capacity of rosehips of some *Rosa* species. Medicines.

[35] Ercisli S. (2007). Chemical composition of fruits in some rose (*Rosa* spp.) species. Food Chem..

[36] Mohammadzadeh N., Ghiasian M., Faradmal J., Dastan D. (2021). Quantitative and qualitative analyses of the constituents of the hydroalcoholic extract of *Quercus infectoria* gall from Kermanshah and evaluation of its antioxidant and antibacterial activities. J. Rep. Pharm. Sci..

[37] Azmaz M., Kilncarslan Aksoy O., Katilmıs Y., Mammadov R. (2020). Investigation of the antioxidant activity and phenolic compounds of *Andricus quercustozae* gall and host plant (*Quercus infectoria*). Int. J. Second. Metab..

[38] Mihaylova D., Georgieva L., Pavlov A. (2015). Antioxidant activity and bioactive compounds of *Rosa canina* L. herbal preparations. Sci. Bull. Ser. F Biotechnol..

[39] Basyigit B., Sağlam H., Köroğlu K., Karaaslan M. (2020). Compositional analysis, biological activity, and food protecting ability of ethanolic extract of *Quercus infectoria* gall. J. Food Process. Preserv..

[40] Xu Y., Tang G., Zhang C., Wang N., Feng Y. (2021). Gallic acid and diabetes mellitus: Its association with oxidative stress. Molecules.

[41] Kang I., Buckner T., Shay N.F., Gu L., Chung S. (2016). Improvements in metabolic health with consumption of ellagic acid and subsequent conversion into urolithins: Evidence and mechanisms. Adv Nutr..

[42] Thiers B (2022 and Continuously Updated). Index Herbariorum: A Global Directory of Public Herbaria and Associated Staff. New York Botanical Garden’s Virtual Herbarium. http://sweetgum.nybg.org/ih.

[43] Šovljanski O., Saveljić A., Aćimović M., Šeregelj V., Pezo L., Tomić A., Cetković G., Tešević V. (2022). Biological profiling of essential oils and hydrolates of *Ocimum basilicum* var. genovese and var. minimum originated from Serbia. Processes.

[44] Šovljanski O., Šeregelj V., Pezo L., Tumbas Šaponjac V., Vulić J., Cvanić T., Markov S., Ćetković G., Čanadanović-Brunet J. (2022). Horned melon pulp, peel, and seed: New insight into phytochemical and biological properties. Antioxidants.

[45] Tumbas-Šaponjac V., Šeregelj V., Ćetković G., Čanadanovic-Brunet J., Đilas S. (2016). Optimization of the composition of the powdered cereal sprouts mixtures. Acta Period. Technol..

[46] Šovljanski O., Saveljić A., Tomić A., Travičić V., Lončar B., Cvetković D., Velićanski A., Pezo L., Ćetković G., Markov S. (2022). Carotenoid-producing yeasts: Selection of the best-performing strain and the total carotenoid extraction procedure. Processes.

[47] Ranitović A., Šovljanski O., Aćimović M., Pezo L., Tomić A., Travičić V., Saveljić A., Cvetković D., Ćetković G., Vulić J. (2022). Biological potential of alternative kombucha beverages fermented on essential oil distillation by-products. Fermentation.

[48] Tomić A., Šovljanski O., Nikolić V., Pezo L., Aćimović M., Cvetković M., Stanojev J., Kuzmanović N., Markov S. (2023). Screening of Antifungal Activity of Essential Oils in Controlling Biocontamination of Historical Papers in Archives. Antibiotics.

[49] Aćimović M., Šovljanski O., Šeregelj V., Pezo L., Zheljazkov V.D., Ljujić J., Tomić A., Cetković G., Čanadanović-Brunet J., Miljković A. (2022). Chemical composition, antioxidant, and antimicrobial activity of *Dracocephalum moldavica* L. essential oil and hydrolate. Plants.

