# Supplementary material for: An In Vitro Evaluation of Robin’s Pincushion Extract as a Novel Bioactive-Based Antistaphylococcal Agent—Comparison to Rosehip and Black Rosehip
Source: Antibiotics (Basel). 2024 Dec 4;13(12):1178. doi: 10.3390/antibiotics13121178 (PMC11672707; doi:10.3390/antibiotics13121178)
Supplement: Supplementary file 1 [file antibiotics-13-01178-s001.zip › antibiotics-3345446-supplementary.pdf]

Supplement material

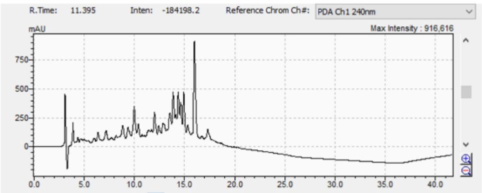

(a) *Robin's pincushion* (240 nm)

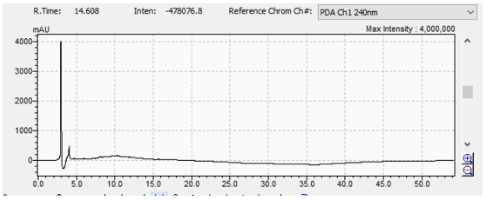

(b) Rosehip (240 nm)

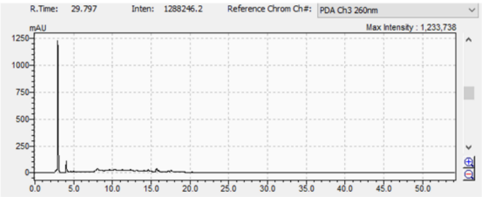

(c) Rosehip (260 nm)

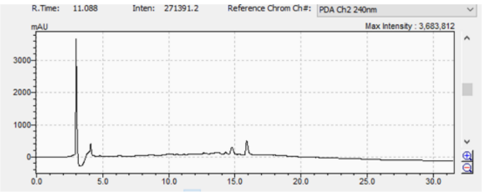

(d) Black rosehip (240 nm)

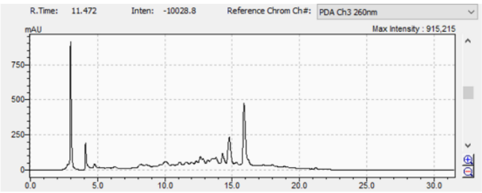

(e) Black rosehip (260 nm)

Supplementary Figure S1. HPLC chromatographs of extracts of (a) Robin's pincushion (240 nm); (b) Rosehip (240 nm); (c) Rosehip (260 nm); (d) Black rosehip (240 nm); (e) Black rosehip (260 nm).
